# Optimal Time Intervals between Pre-Operative Radiotherapy or Chemoradiotherapy and Surgery in Rectal Cancer?

**DOI:** 10.3389/fonc.2014.00050

**Published:** 2014-04-07

**Authors:** Bengt Glimelius

**Affiliations:** ^1^Department of Radiology, Oncology and Radiation Science, Uppsala University, Uppsala, Sweden

**Keywords:** rectal cancer, radiotherapy, chemoradiotherapy, surgery, time interval, tumor regression

## Abstract

**Background:** In rectal cancer therapy, radiotherapy or chemoradiotherapy (RT/CRT) is extensively used pre-operatively to (i) decrease local recurrence risks, (ii) allow radical surgery in non-resectable tumors, and (iii) increase the chances of sphincter-saving surgery or (iv) organ-preservation. There is a growing interest among clinicians and scientists to prolong the interval from the RT/CRT to surgery to achieve maximal tumor regression and to diminish complications during surgery.

**Methods:** The pros and cons of delaying surgery depending upon the aim of the pre-operative RT/CRT are critically evaluated.

**Results:** Depending upon the clinical situation, the need for a time interval prior to surgery to allow tumor regression varies. In the first and most common situation (i), no regression is needed and any delay beyond what is needed for the acute radiation reaction in surrounding tissues to wash out can potentially only be deleterious. After short-course RT (5Gyx5) with immediate surgery, the ideal time between the last radiation fraction is 2–5 days, since a slightly longer interval appears to increase surgical complications. A delay beyond 4 weeks appears safe; it results in tumor regression including pathologic complete responses, but is not yet fully evaluated concerning oncologic outcome. Surgical complications do not appear to be influenced by the CRT-surgery interval within reasonable limits (about 4–12 weeks), but this has not been sufficiently explored. Maximum tumor regression may not be seen in rectal adenocarcinomas until after several months; thus, a longer than usual delay may be of benefit in well responding tumors if limited or no surgery is planned, as in (iii) or (iv), otherwise not.

**Conclusion:** A longer time interval after CRT is undoubtedly of benefit in some clinical situations but may be counterproductive in most situations. After short-course RT, long-term results from the clinical trials are not yet available to routinely recommend an interval longer than 2–5 days, unless the tumor is non-resectable at diagnosis.

## Introduction

In rectal cancer, radiotherapy (RT) or chemoradiotherapy (CRT) is extensively used pre-operatively to decrease the risk of local failure in resectable tumors by sterilizing microscopic tumor foci not removed by the surgeon and to allow radical surgery in non-resectable or difficult to resect tumors. RT/CRT is also used to increase the chances of sphincter-saving surgery and to omit or limit the extent of surgery. For a successful outcome, the size or the stage of the rectal tumor must be decreased in the three last mentioned clinical situations, but not in the one mentioned first where it is sufficient to influence eventual cells remaining after surgery so that they are no longer clonogenic. An interval between the end of the RT/CRT is required if tumor regression is required. During the interval, the acute tissue reaction from the radiation, potentially increasing the risk of surgical complications, also subsides.

**Figure 1:**
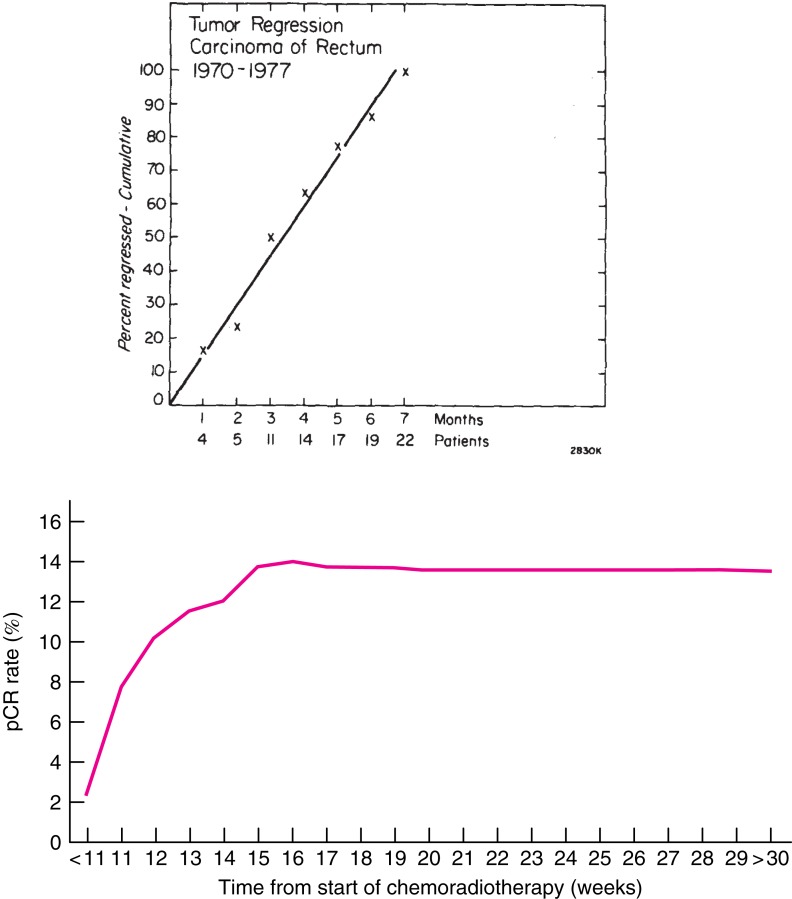
**Time course of complete regression of rectal cancer after radiation therapy**. Above: Results from 22 patients with mobile tumors treated by radiation alone, 50 Gy in 20 fractions in 4 weeks at PMH, Toronto, Canada, for whom detailed observations of complete regression were available (1). The primary tumor was controlled in 21 (38%) of 56 patients with mobile tumors and in only 6 (9%) of 67 fixed tumors. Below: Results from 1593 patients treated with CRT and selected from the Dutch surgical colorectal audit (4). The *x*-axis in the figure above refers to the interval from the end of RT to surgery (months) and below from start of CRT (weeks). The number of patients treated with longer intervals is few and these proportions are thus very uncertain. All data are collected retrospectively and reasons for the long delay are not known. In spite of this, the figures illustrate that tumor regression in rectal cancer is slow. Reprinted with permission from Wolters Kluwer Health (above) and John Wiley and Sons (below).

Radiotherapy was early developed in squamous cell carcinomas from the head and neck region for the same reasons as used in rectal cancer, visually to decrease the risk of loco-regional failure, to allow surgery, and to preserve the organ function. The RT resulted in an acute tissue reaction that healed in a couple of weeks, delaying surgery for about a month. Squamous cell carcinomas regress quite rapidly. In contrast, when RT was used for adenocarcinomas, like rectal cancer, it was at an early stage observed that tumor regression was much slower and did not become maximal until after several months (Figure 1) (1, 2). A median tumor volume-halving time of 14 days has later been reported (3).

When RT was applied pre-operatively to rectal cancer it was usually not required to achieve maximum tumor regression and the time interval was decided by the acute radiation reaction to wear off. Thus an interval of 3–5 weeks was chosen. As an alternative to conventionally fractionated RT, a short-course schedule was developed for use in resectable tumors. Since there was no need for tumor regression, surgery was performed immediately, or within 3–5 days from the last radiation fraction (5). If the tumor at surgery was non-resectable, the abdomen had to be closed. The experience was then that the tumor regressed and could be resected at a later stage. This experience led to the use of the short-course schedule in elderly or co-morbid patients with non-resectable tumors, when the reference treatment CRT was not considered tolerable (6–8).

There is an increasing, clearly articulated view among clinicians and scientists to prolong the interval to achieve maximal tumor regression. A complete clinical response (cCR) and, if the patient is operated upon, a complete pathological response (pCR, i.e., ypT0N0) have developed as important prognostic factors indicating low recurrence risks (9–12). This was known decades ago (2, 13). Recent evidence was subject to a systematic overview including 3105 patients with 484 pCRs from 14 studies and reported improved disease-free survival (DFS) at 5 years (adjusted HR 0.54, 95% CI 0.40–0.73) (14). In another systematic review of 16 studies with 3363 patients (15), a pCR was associated with fewer local recurrences (odds ratio, OR = 0.25), less distant failures (OR = 0.23), and improved overall survival (OS) (OR = 3.28) and DFS (OR = 4.33). pCR is now considered a relevant endpoint after pre-operative therapy, indicating a prognostically favorable tumor biological profile with less risk of recurrence and better survival, although its use as an endpoint in clinical trials has been questioned (16). Partial regression is likely also relevant (17), although not universally found (18). The evaluation of pathological tumor regression is non-standardized and not reproducible (19). Evaluations using MRI may be more reproducible and are available prior to surgery (20). The possibilities to achieve CR are behind the growing interest in organ-preservation, i.e., to omit surgery or limit surgery to a local excision (21–24).

The observation of favorable prognosis if cCR/pCR is achieved has led many investigators to increase the radiation dose to the tumor center (25, 26), to prolong the interval to surgery, to add chemotherapy in the interval (27–30), or to start with chemotherapy (31–33). All approaches intuitively seem attractive, and to prolong the interval can be achieved without any increasing costs. Since there also is an impression that complications to surgery decrease with longer time intervals, this has been adopted by many and intervals much longer than the originally used 3–6 weeks have been applied (3, 14, 34).

This article will critically review in what clinical situations more down-sizing/down-staging can be beneficial and to review whether a longer than usual interval in situations where tumor regression is not of any benefit, actually can be deleterious. The presentation will include both the time interval after short-course RT and after conventionally fractionated CRT. The aim was to include all relevant published literature, but systemic overviews based primarily only upon retrospective studies are very difficult to get complete. In the literature searches performed in August and December 2013, several relevant articles were identified that was not included in an article claimed to be systematic and published online during the preparation of this review (35).

## Pre-Operative Radiotherapy to Improve Local Control and Survival

In rectal cancer therapy, the pre-treatment division into three groups, the good–bad–ugly concept (36, 37) has been useful in the selection of initial therapy, and included in recent guidelines/consensus documents (38–41). In early/good tumors, the risk of local failure is so small, provided proper staging, and surgery is done, that it is not considered appropriate to reduce it further with pre-operative RT. There is no consensus on how high the risk could be, but a risk of at the most 5–8% is reasonable (42). The patients should participate in this decision, balancing the morbidity from a local failure and the added morbidity to surgery from the pre-operative RT, but studies on patient preferences in rectal cancer therapy are few and inconclusive (43–45). In one study, an absolute 10% difference was required for half of the 50 patients to accept the morbidity from CRT (45). In the intermediate/bad group, the risk of local failure after surgery alone is higher or above 8–10%, and the 50–70% reduction from pre-operative RT/CRT is meaningful. The term intermediate/bad is preferred for this group, even if most trialists have called these tumors “locally advanced” (42). In the locally advanced/ugly group, surgery alone must either be extensive to achieve local radicality (circumferential resection margin negativity, crm−) or will result in high local failure rates (≥25%). It is only in this group that tumor regression is required to achieve high probability of local control after surgery. The cT3 mrf+ (clinical stage T3 with mesorectal fascia involvement <1 mm) and most cT4 tumors are considered “ugly.” In contrast, it is not possible to clearly separate the “good” and “bad” tumors from each other using T and N stage only since many other factors like distance form the anus, the size of the mesorectum (varies according to level, direction, sex, and between individuals), presence of vascular invasion, and the skill of the multidisciplinary team are important. The majority of cT1-2 tumors and many cT3 mrf-tumors in middle and high rectum belong to the “good” group. Node positivity on MRI is not necessarily sufficient to consider that the risk of local recurrence is sufficiently high, requiring pre-operative RT/CRT.

The risk of systemic relapse is not identical to the risk of local relapse, although a strong overlap is seen (36, 46). Node positivity and extramural vascular invasion are likely more important for systemic dissemination than local relapse.

## Time Interval after Short-Course Radiotherapy to Surgery

The “ideal” short-course schedule, five fractions of 5 Gy Monday through Friday with surgery the coming Monday or Tuesday results in an interval of less than 10 days. Retrospective analyses of randomized trials have not detected any differences in tumor control if the RT started on another weekday than Monday, resulting in a weekend-break during the RT (47, 48). This schedule, developed to be used in tumors presently designated intermediate/bad staged by MRI (originally resectable tumors, excluding early, polypoid cancers (5) to decrease local failure rates, was not supposed to result in down-staging (49, 50). A retrospective analysis of Swedish data showed that down-staging was seen when the interval was longer than 10 days from the first RT fraction (47). Starting with anecdotal experiences of patients who had received 5Gyx5 and where the tumor was found non-resectable at surgery the coming week but where it could be radically resected several weeks later, several groups have now reported favorable outcomes after short-course RT with a delay in elderly and co-morbid patients with non-resectable rectal cancer not tolerating conventional CRT (6–8). This has lead to a renewed interest in exploring short-course RT with a delay also in younger patients with less advanced cancers. The Stockholm III trial randomly tested short-course RT with immediate surgery (reference treatment) against short-course RT with surgery delayed, initially for 4–6 weeks, but more and more for 6–8 weeks or long-course RT (2Gyx25) in intermediate rectal cancers. In an interim analysis after inclusion of 303 patients, all treatment arms were feasible (51). The reference treatment resulted in more toxicity after surgery than the two other arms, but this disappeared when patients with a delay from the start of RT to surgery above 10 days, violating the protocol, were excluded. Thus, based upon this subgroup analysis, supported by subgroup analyses of the Stockholm I + II studies (52) and the TME study (48, 53), surgery after 5Gyx5 should be performed within 11 days from the start of RT, or delayed for several weeks in order to minimize surgical morbidity and mortality. Even if similar experience has been reported by others (54, 55), the conclusion is based upon retrospective studies, and should be interpreted cautiously. In an analysis of the Stockholm III trial after 657 randomized patients (585 analyzed) (56) and in a validation set to the Dutch study (48), the increased toxicity after a slight delay was no longer seen. It can be speculated that if surgeons know about the risk of increased toxicity with a slight delay to surgery, it is no longer a risk factor. Impaired leukocyte response after surgery can be a reason for the increased risk of toxicity, particularly if surgery is done between 10 to about 21 days after start of RT (52, 55, 56).

In a Polish study, 154 patients with intermediate rectal cancers were randomized to 5Gyx5 with surgery either 7–10 days or 4–5 weeks after the end of RT (57). More down-staging was seen, but the rate of sphincter-preservations and curative resections did not increase. More local recurrences were seen in the group randomized to a longer time interval (9 vs. 1%, not statistically significant), but fewer systemic relapses. More down-staging was also seen in the Stockholm III study in an evaluation of the first 400 patients randomized to short-course RT with delayed surgery compared to immediate surgery (pCR in 13 vs. 2%, *p* < 0.001) (58). Similar findings were reported in a retrospective evaluation of 67 Dutch patients (59).

To summarize (Table 1), 5Gyx5 results in down-staging that is apparent in large patient series already after 11 days after the first radiation fraction. It becomes clinically and radiologically apparent in individual patients after 3–4 weeks, permitting radical surgery in patients with initially non-resectable tumors. A pCR is seen in 10–15% in tumors belonging to the intermediate/bad and locally advanced/ugly groups. Whether a delay to surgery changes the oncologic outcome will not be known until results from the Stockholm III trial are available (in 2015). Delaying surgery for 4 weeks or more does not result in more surgical morbidity than if surgery is done immediately. Actually, it may be less. On the other hand, several retrospective analyses have reported less toxicity if surgery is done, as originally intended, within the first few days after the fifth radiation fraction. Thus, a longer than intended interval should be avoided. If surgery can not be performed within the first few days (not within the first week), it should be delayed for at least 3–4 weeks, letting the acute radiation reaction subside.

**Table 1:** **Relevance of the time interval from short-course RT to surgery with level of evidence**.

Interval (from the last 5 Gy fraction)	Surgical morbidity		Tumor regression		Oncologic outcome	
2–4 days (reference)	Known (limited)	I^b^	0	I	Known (50–70% reduction in LR)	I
5–~15 days	Increased	III	Detectable	III	Likely the same	III
20+ days^a^	Known (limited)	II	Yes, pCR 10–15%	I	Not yet known, the Stockholm III trial may give information	IV

*Late toxicity from short-course RT will likely be the same irrespective of the time interval*.

*^a^No information about surgical morbidity or tumor regression is available if the interval is only 3–6 weeks or longer*.

*^b^Level of scientific evidence according to GRADE (Roman numbers, I–IV where I = high quality, II = moderate quality, III = low quality, and IV = very low quality) (60)*.

## Time Interval after Long-Course Chemoradiotherapy to Surgery

### Pathological complete remission/tumor regression

Numerous studies have shown a correlation between the time interval after RT to 45–50 Gy with concomitant fluoropyrimidine (CRT) and the extent of tumor regression. A meta-analysis of 13 studies, including 3584 patients found that an interval longer than the “conventionally” 6–8 weeks resulted in more pCRs (relative risk 1.42, absolute increase from 14 to 20%) (35). In the largest study, 1593 patients from 92 Dutch hospitals who underwent pre-operative CRT between 2009 and 2011 (4) had the highest chance of pCR if the CRT-surgery time interval was about 11 weeks (15–16 weeks after start of CRT). Median interval was 9–10 weeks, likely reflecting the common belief that a longer interval than used in the randomized trials testing the value of pre-operative CRT (61–64) is advantageous. The rate of pCR increased from 1 to 2% in those operated within the first 5–6 weeks up to 14% if operated after 11 weeks, with no apparent further increase beyond that time (Figure 1).

Of several other retrospective analyses (3, 34, 65–67), not included in the meta-analysis (35), only one (3) revealed a significant association. Lymph-node retrieval was in one study lower after neo-adjuvant therapy with an inverse correlation between the yield and the time from (C)RT (68). This may influence post-operative decision-making (69).

### Other endpoints than tumor regression

Besides the established finding that tumor regression increases with the duration of the interval, less is known about whether surgical morbidity is decreased and oncologic outcomes improved (sphincter- or organ-preservation, local recurrence rate, and survival). The meta-analysis (35) extracted information about these outcomes from 6–8 of the 13 trials, including about 1300–2700 patients. No analyzed outcome differed according to time interval. Thus, OS (RR = 0.85, 95% CI 0.5–1.43) and DFS (RR = 0.81, 95% CI 0.58–1.12) were not different. No difference was seen for R0 resection and sphincter-preservation rates. Wound complications and anastomotic leak events were also similar (RR = 0.83, *p* = 0.27).

More perineal wound complications [OR = 0.97 (95% CI 0.95–0.99)] and anastomotic leakage [OR = 0.97 (95% CI 0.94–1.00)] were seen with shorter interval (by 1 week) in a study that included 189 patients (34). Interval was not related to local recurrence, metastasis, or death. This study could not detect any relation between interval (median 10 weeks, range 1–30) and pathological outcome. No other study has reported any detectable influence on the post-operative course.

With few exceptions, none of the other studies has reported any influence on oncologic outcome. In a retrospective analysis of 177 patients, more pCRs (31 vs. 17%) and fewer local recurrences (1 vs. 11%, *p* = 0.04) were seen with an interval above 8 weeks after CRT, but no difference in post-operative morbidity was detected (70). Similarly, in a study including 132 patients, no difference in morbidity was seen in patients operated after 7 weeks, whereas more pCR/near pCR and improved DFS (*p* = 0.05) were seen (71). In yet another analysis of 397 patients, delaying surgery after CRT from 4–6 to 6–8 weeks did not increase tumor response, sphincter-preservation rate or decrease morbidity or local recurrence rate (72). In the study, the anastomosis-related complication rate was the same (4–6%) irrespective of the interval. In a study, evaluating 33 patients treated with CRT including irinotecan, a 10- to 14-week interval did not influence post-operative morbidity (or pCR-rates) compared to 4–8 weeks (73). In a study (65) of about 160 patients treated with pre-operative CRT (47 Gy with Xelox), an interval between 40 and 70 days was accompanied by more alterations in the tumors, but unaccompanied by changes indicating more favorable outcomes compared to an interval of 21–40 days. Post-operative complications were the same. In an analysis of 102 patients treated with pre-operative RT (45Gy x25) alone, the recommendation was to operate as soon as possible, unless sphincter-preservation could be possible (66). No difference in the proportion of patients with an early tumor (pT0-2N0) or in survival was seen whether the RT-surgery interval was shorter or longer than 6 weeks. A longer time from diagnosis to surgery negatively influenced metastasis-free survival (≥16 weeks, OR = 2.05, *p* = 0.05). In another study of 88 patients, no differences were seen in pCR-rate or down-staging whether the interval was shorter or longer than 28 days (67). A pCR favorably influenced prognosis, as reported by many others, but the study did not report whether the CRT-surgery interval influenced prognosis or complication rates.

Two studies have used a fractionation schedule that differs from all other studies. In the randomized Lyon R90-01 trial (74), pre-operative RT (39 Gy in 13 fractions) was randomly followed by surgery within 2 weeks or after 6–8 weeks. More clinical and pathological down-staging was seen in the long-interval group. Morbidity, local relapse, survival, or sphincter-preserving surgery rates did not differ. In a retrospective evaluation of 250 patients treated with hyperfractionated accelerated RT (41.6 Gy/26 fractions × BID) with planned immediate surgery, patients who had an interval of >5 days had better OS (69 vs. 47%, *p* = 0.002), DFS (62 vs. 41%, *p* = 0.0003) but not local control (93 vs. 90%) (75). The authors speculate if it is a matter of days?

To summarize (Table 2), long-course CRT to 45–50.4 Gy results in down-staging that is more pronounced with longer CRT-surgery interval, at least up to an interval of 10–11 weeks. This is most easily appreciated as increased pCR-rates (35). Although reported in one study (34), there is no evidence that a longer interval within the time-span 4–12 weeks results in less morbidity. Surgery within the first 3–4 weeks is not recommended due to the acute radiation reaction. There is very limited experience with intervals beyond 12 weeks, unless chemotherapy has been given in the interval (see below). There is a fear that radiation-induced fibrosis will make surgery more difficult, but this has not been substantiated in any report. It is possible that chemotherapy in the interval may delay or prevent the development of fibrosis. The evidence that the CRT-surgery interval (4–12 weeks) influences oncologic outcome is virtually lacking. Thus, there appears to be no reason to prolong the interval from 4–6 to 6–8 weeks or more, as suggested, in tumors upfront considered resectable to improve local control rates or survival.

**Table 2:** **Relevance of the time interval from long-course CRT to surgery on different outcomes**.

Outcome	Effect of a longer compared to a shorter interval	Reference	
		Systematic overview (35)	Other^a^
pCR/regression	Increased (OR 1.42)	Yes	(66)
Surgical morbidity, predominantly wound healing, anastomotic leakage	Unchanged (RR = 0.87) (slightly decreased in one study)	Yes	(34, 65, 70, 72, 76)
Overall survival	Unchanged (RR = 0.85)	Yes	(66, 76)
Disease-free survival	Unchanged (RR = 0.81)	Yes	(34, 66, 76)
Local recurrence	Unchanged		(66, 70, 72)
Sphincter-saving surgery	Unchanged		(65, 72)
Lymph-node retrieval	Decreased		(68)

*^a^Only references not included in the systematic overview (34) are listed*.

## Increasing the Dose and/or Adding Chemotherapy in the Interval

In patients with non-resectable tumors or with resectable tumors but unfit or unwilling to undergo surgery, the radiation dose has since decades been increased to increase the possibilities to local and total cure without surgery (2, 13). CRs were seen and a small but definite proportion was cured (13). This has also recently been reported from a Polish group; large fixed or locally recurrent tumors could be controlled with acceptable risks of late bowel toxicity (77).

The randomized Lyon R96-02 trial revealed more sphincter-preservation when three contact x-ray boosts (20–35 Gy) were added to external-beam RT (39Gyx13) (78). Eighty-eight patients were included. After 10 years, there was no difference in local recurrence rates (about 10%) and OS (79).

In a study including 222 patients treated with external-beam CRT and brachytherapy, a dose–response modeling revealed a dose–response relationship, both for pCR [D_50_-92 (95% CI 19–145)Gy] and for tumor regression [D_50_-72 (95% CI 65–94)Gy] (80). Thus, comparably high doses are required to obtain a 50% probability of a major response in locally advanced rectal cancers. The time interval was not considered. In the randomized study by the same group, a brachytherapy boost of 10 Gy in two fractions after CRT to 50.4 Gy did however not increase pCR-rates (18% in both arms, *n* = 248) (81). Major responses were increased (44 vs. 29%, *p* = 0.04).

Habr-Gama and co-workers in Brazil have since the early 1990s explored the potential for organ-preservation, i.e., in a sense prolonging the interval to surgery indefinitely in patients with tumors regressing completely (21). In 71 (28% of 265 patients) with cCR after 12 months, only two local recurrences were seen. Their studies will not directly throw more light on the most optimal (C)RT-surgery interval, since their ambition has been to avoid surgery whenever possible. They do however indirectly bring further light on this issue. More recently, the radiation dose has increased from traditionally 45–50 Gy to 54 Gy with chemotherapy (three cycles of 5-FU/leucovorin after the CRT) given in the interval. A high CR rate (65, 48% clinical, 17% pathological) was seen in 34 patients (27). Toxicity was acceptable. In a more recent publication (82), 47 (68%) patients out of 70 starting therapy had cCR at 10 weeks. During the first year, eight (17%) patients recurred locally. Of those with cCR at 12 months, four (10%) did so. The authors believe that this dose-intensification/interval prolongation has resulted in improved results with half of the patients never requiring surgery. Since patient selection may be as relevant as therapy intensification, it is difficult to make firm conclusions. Garcia-Aquilar (29) has similarly extended the interval from the standard of 6–11 weeks, filling the extra gap with chemotherapy. They reported only a small increase in the pCR-rates (18–25%). Post-operative complications did not increase.

Repeated PET–CT examinations were used to explore the optimal interval to evaluate tumor regression (83). Ninety-one patients selected to treatment with CRT for potential organ-preservation had FDG-PET at baseline, after 6 and 12 weeks. The mean maximal SUV-uptake decreased from baseline to 6 weeks. This decrease was not predictive for response, whereas a decrease from 1 to 3 h at the 6 weeks investigation was (dual time-point imaging). About half of the patients showed a further slight decrease from 6 to 12 weeks, whereas half did not; rather, a slight increase in SUV_max_ was seen (poor responders). A sustained cCR was rarely seen in the group of poor responders. Although the relevance of these changes in SUV_max_ uptake is not known, they may indicate that for about half of the patients, tumor repopulation starts after about 6 weeks.

Higher radiation doses have also in several other patient series been associated with better tumor response, although often confounded by more intensive chemotherapy (84–87). Thus, it appears rationale to increase the dose to the tumor if it is important to achieve more complete or major responses. This may be relevant in peripheral parts of the tumor if the aim is to achieve an R0 resection in initially non-resectable tumors with overgrowth to adjacent structures (88), or centrally if the aim is sphincter- or organ-preservation; otherwise not.

## Discussion

The knowledge about the relevance of the time interval from the start (or the end) of pre-operative (C)RT is chiefly based upon retrospective analyses of hospital-based series and a few population-based and randomized studies. Since randomized studies directly comparing two intervals without making any other intervention are few in numbers, the level of scientific evidence is formally poor but still based upon experiences collected during decades. The three randomized studies (51, 57, 58, 74), all reported more clinical and pathological down-staging with longer intervals to surgery. Since this was also seen in a meta-analysis of several retrospective studies (35), and is logical, there is no doubt that a longer time interval to surgery up to about 8–12 weeks after a radiation dose equivalent to about 45–50 Gy in 4–5 weeks will result in more tumor regression.

The randomized studies also showed that delaying surgery for 4–8 weeks is safe (51, 57, 58, 74). Actually, it is possible that surgical morbidity after short-course RT is less if surgery is delayed than if performed immediately, but this is confounded by patients who were operated upon after a brief delay of only a few days, revealing increased surgical morbidity (51). There may be a time-period after short-course RT during which surgery should not be performed (48, 52, 54, 55), even if there are indications that knowledge about the increased morbidity seen when surgery is performed between 10 and 20 days after the first 5 Gy fraction can be handled (48, 56). Delaying surgery after long-course CRT also appears safe. With the exception of one study that actually showed less complications associated with longer delay (34), no study reported more complications within the time-span of 4–12 weeks. Even if patients have had surgery beyond 12 weeks, they are so few that no conclusions can be made.

In patients responding with regression to RT, it is likely safe from an oncologic perspective to delay surgery for at least several weeks. Withers and Haustermans (89) also state that “there is no tumor-related necessity for early post-radiation surgery.” The standard doses used pre-operatively usually mean that the tumor cells are not viable clonogens to metastasize, although caution must be made concerning the great heterogeneity in response. The increase in FDG-PET uptake between 6 and 12 weeks in half of the predominantly early tumors treated for organ-preservation in Brazil (83) may be an indication of repopulation. Thus concerns must be expressed in tumors not responding well to the radiation. There are no solid data telling that regrowth of the irradiated primary tumor or pathological lymph nodes has started during a time interval of up to 10–12 weeks, but this has not been extensively studied. In head- and neck-cancer, repopulation starts soon after the radiation has stopped, with a proliferation rate equal to 0.75 Gy/day (90). The knowledge about the corresponding value for rectal cancer is limited (91), although it may be longer than generally perceived. Rectal cancers are also heterogeneous in their proliferative activity and this influences radiation sensitivity (92, 93).

Another concern, never expressed in any of the articles presenting results after different CRT-surgery intervals, is growth of disseminated tumor cells not irradiated, although discussed by Withers and Haustermans (89). Adjuvant post-operative chemotherapy has in the rectal cancer trials not shown the same clear benefit as in the colon cancer trials (38, 94). When metastatic, colon, and rectal cancers respond similarly to chemotherapy and primary tumor site is not predictive of response or survival. There are thus no tumor biological apparent reason to believe that there should be a discrepancy in the adjuvant situation, although it is known that on a group level, differences in molecular properties that could indicate chemotherapy resistance exist between colon and rectal cancers (95). There are however many differences between the trials that could explain this apparent discrepancy. Since the number of trials and the number of patients in the colon cancer trials are many more than in the rectal cancer trials, it may be a power problem. The pre-operative RT/CRT could not really influence the sensitivity of already disseminated cells, but it could decrease tolerability to the treatment and thus result in less gain. Further, in the colon cancer trials revealing significant gains from adjuvant chemotherapy, patients have been treated for at least 6 months, whereas in most adjuvant rectal cancer trials, treatment has only lasted for 4 months. We have presently no knowledge about whether the efficacy of a 4-month treatment with 5-FU/leucovorin is as efficient as 6–8 months. This is neither true for oxaliplatin-based combinations, although trials are presently comparing 3 and 6 months treatment (96). Finally and potentially most important, the adjuvant colon cancer trials showing significant survival gains all required that the adjuvant treatment started within 5–6 weeks after surgery. This has not been the case in the rectal cancer trials.

Retrospective analyses have indicated that time to start of adjuvant chemotherapy is relevant (97), but selection bias can not be excluded. Some have indicated that prognosis is worse if the delay is longer than 8 weeks, others if it is longer than 12 weeks. The adjuvant therapy in colon cancer is proven, and valued by doctors and patients, but it is still limited and it is quite possible that a few weeks prolongation may mean proliferation of tumor cells so that they are no longer possible to eradicate by the chemotherapy. Then, a delay from about 5–6 to 8–10 weeks may be relevant. In colon cancer patients, surgery is usually performed within a few weeks and the adjuvant treatment can often start 5–6 weeks later, i.e., a delay of at the most 2 months from diagnosis. In rectal cancer, planning and delivery of RT, particularly if long-course (C)RT, waiting for surgery and for the post-operative recovery may mean that the subclinical deposits do not receive any efficient systemic chemotherapy until several months later. May be this is the clue to the discrepancy in efficacy of adjuvant chemotherapy between colon and rectal cancers. This calls for neo-adjuvant chemotherapy, and trials are ongoing (98). This, however, further tells that any prolongation of time from RT/CRT to surgery in a tumor that is primarily resectable can only be deleterious. Statements like “waiting for the highest degree of pathological response is clinically relevant as it increases the chance of R0 resection” (4) make no sense. The cited statement probably reflects the opinions of many clinicians treating rectal cancer patients. In resectable tumors, the chance of an R0 resection with proper surgery is not increased even if the pathological response gets better, it should be 100% anyhow. The cell kill is already done and any prolongation of the interval may only diminish the chance that adjuvant chemotherapy will eradicate all cells. Tumors not responding well to the (C)RT may also have started to repopulate in such a way that it may metastasize, even if not believed (89).

Organ-preservation, or to prolong the interval indefinitely, appears from the ongoing debate to be a new phenomenon, starting with the Habr-Gama publications in 2004 (21, 99, 100). There has for decades been a desire to avoid removal of the sphincters and to avoid major surgery in risk groups (1, 2), and RT has been one tool to permit this. In my early carrier as an oncologist with interest in rectal cancer, we had in the late 1970s-early 1980s many elderly patients above 75–80 years whom it was too risky to operate (today they are usually above 85–90 years) and RT or CRT was used to locally control the disease. This was successful in some, although most still suffered, sometimes severely from the local tumor burden after a period of good relief (101). The results clearly showed the heterogeneity in response among rectal cancers. The radiation doses given then were as high as they can be today even if the tumor could be not be precisely located and delivery not so conformed as today. Thus, the expected toxicity today is less (77). A fluoropyrimidine was added as radiation sensitizer already in those days when the wish was to reach as high cell kill effect as possible, although the acute toxicity often prevented its use throughout the treatment in elderly patients. Despite more than 30 years of research, no more effective radiosensitizer is available. Later randomized trials have shown that CRT is more effective than RT (62–64), but the improvement is not marked, although again valued by doctors and patients, and unfortunately comes at a price with more acute and also late toxicity (102, 103). The wish to obtain cCR has prompted many doctors to prolong the interval to planned surgery even if there is no or limited chance to avoid surgery or increase sphincter-preservation. That prolongation is of no advantage for the patient, and should be avoided since it can only be potentially deleterious. Until we know more from trials, it is better to keep the (C)RT-surgery interval as short a possible, allowing the acute radiation reaction not to start (short-course) or to subside (short- and long-course).

## Conflict of Interest Statement

The author declares that the research was conducted in the absence of any commercial or financial relationships that could be construed as a potential conflict of interest.
